# High-Performance Ultraviolet Light Detection Using Nano-Scale-Fin Isolation AlGaN/GaN Heterostructures with ZnO Nanorods

**DOI:** 10.3390/nano9030440

**Published:** 2019-03-15

**Authors:** Fasihullah Khan, Waqar Khan, Sam-Dong Kim

**Affiliations:** Division of Electronics and Electrical Engineering, Dongguk University, Seoul 100-715, Korea; fasihullah.khan@dongguk.edu (F.K.); waqarkyz@gmail.com (W.K.)

**Keywords:** high-responsivity, ultraviolet photodetectors, nano-scale fin isolation, wide-band gap semiconductors, ZnO nanorods, two-dimensional electron gas, visible-blind

## Abstract

Owing to their intrinsic wide bandgap properties ZnO and GaN materials are widely used for fabricating passive-type visible-blind ultraviolet (UV) photodetectors (PDs). However, most of these PDs have a very low spectral responsivity ***R***, which is not sufficient for detecting very low-level UV signals. We demonstrate an active type UV PD with a ZnO nanorod (NR) structure for the floating gate of AlGaN/GaN high electron mobility transistor (HEMT), where the AlGaN/GaN epitaxial layers are isolated by the nano-scale fins (NFIs) of two different fin widths (70 and 80 nm). In the dark condition, oxygen adsorbed at the surface of the ZnO NRs generates negative gate potential. Upon UV light illumination, the negative charge on the ZnO NRs is reduced due to desorption of oxygen, and this reversible process controls the source-drain carrier transport property of HEMT based PDs. The NFI PDs of a 70 nm fin width show the highest ***R*** of a ~3.2 × 10^7^ A/W at 340 nm wavelength among the solid-state UV PDs reported to date. We also compare the performances of NFI PDs with those of conventional mesa isolation (MI, 40 × 100 µm^2^). NFI devices show ~100 times enhanced ***R*** and on-off current ratio than those of MI devices. Due to the volume effect of the small active region, a much faster response speed (rise-up and fall-off times of 0.21 and 1.05 s) is also obtained from the NFI PDs with a 70 nm fin width upon the UV on-off transient.

## 1. Introduction

Ultraviolet (UV) light detection with high responsivity is of great interest, due to its promising applications in pathology [1], water treatment, safety, and defense-technologies [2]. In most commercialized systems, UV detection has been realized by photomultiplier tubes (PMTs), thermal detectors, Si or Ge based photodetectors (PDs), and charge-coupled devices (CCDs). However, these detection methods pose various problems for practical applications. For example, PMTs exhibit fragile vacuum-tube construction and require a high-voltage bias. Moreover, PMTs are sensitive to the magnetic-field; therefore, they need to be shielded from ambient light. Si based PDs or CCDs are one of the most commonly used solutions, due to their highly matured process technology [3]. Recently, n-Si/p^+^-B photodiodes of a very high sensitivity and stability have been demonstrated, but they still exhibit responsivity in the order of ~10^−1^ A/W [4]. Many attempts have been made to improve the limited responsivity of Si PDs working in linear mode. Among them, avalanche-mode PDs are now very popular, and single-photon avalanche diodes are also used to obtain high-gain responsivity [5]. However, the use of stop-band filters for visible light reduces the quantum efficiency of these devices in the UV range. 

In recent years, the nanostructures of wide bandgap (WBG) materials, such as diamond, ZnO, III-nitride, and SiC based UV PDs have attracted tremendous research interest for their many advantages [6]. For instance, they are capable of room temperature (RT) operation and have intrinsic optical transparency (visibly-blindness) in the visible spectral range. Furthermore, they have a low thermal conductivity, high breakdown field, and stability at elevated temperatures. In spite of these promising advantages, the WBG semiconductors have very low electron mobility. Even though noble heterostructures with a very high electron mobility of ~10^6^ cm^2^/V·s using a material system such as MgZnO/ZnO [7] have been reported, most of the passive PDs fabricated using conventional WBG semiconductors have very low spectral responsivity [8]. Moreover, the response speeds of PDs based on ZnO or GaN are very slow in general because the photoresponse characteristics depend on the well-known bottle-neck chemisorption process of oxygen at the surface of such materials [9].

Zinc oxide (ZnO) nanorod (NR) based UV PDs [10,11,12,13,14,15] have shown promising results in terms of response speed and spectral responsivity [16]. Significant research effort has been made to improve the performance of the PDs, either by improving the crystalline quality of NRs or by utilizing the composite coaxial structure of ZnO with other materials, such as copper oxide [17] and graphene [18]. However, most of the passive type PDs reported to date exhibit very slow response speed (tens of seconds) and low responsivity (~hundreds of A/W). Recently, an active-type UV PD using the ZnO NR-gated AlGaN/GaN high electron mobility transistor (HEMT) structure has been attempted as a part of an effort to attain a breakthrough in responsivity (~10^5^ A/W) and to obtain a relatively fast response speed [19]. However, due to the very high dark current of the device, the HEMT based PD showed a relatively low on-off current ratio of ~3.

In this study, we demonstrate high-responsivity UV PDs based on the ZnO NR-gated AlGaN/GaN HEMT structure with nano-scale fin isolation (NFI). ZnO NRs act as the floating gate while the UV driven chemisorption process of oxygen at the surface of ZnO NRs [20] controls the conduction of the underlying two-dimensional electron gas (2-DEG) channel. The 2-DEG present at the interface of the AlGaN (barrier) and GaN (channel) layers is due to polarization induced electric potential in the heterostructure [21]. In the NFI PD structure, ZnO NRs surround the channel in the gate area. Consequently, the carriers in the 2-DEG channel are confined along the channel and in perpendicular direction to the interface. Therefore, under dark conditions, the 2-DEG channel is fully depleted due to negative surface potential generated via oxygen adsorption at the surface of ZnO NRs [9]. This structure resembles enhancement-mode (normally-off) AlGaN/GaN fin-shaped field-effect transistors (FINFETs) [22], demonstrating extremely broad transconductance and excellent off-state characteristics. In this study, the performance of the NFI PDs is compared to conventional mesa isolation (MI) AlGaN/GaN HEMT based PD of ~100 × 40 µm^2^ active area. We examine various photoresponse characteristics of NFI and MI devices and investigate how structural differences influence their performance.

## 2. Device Fabrication and Characterization

**Figure 1:**
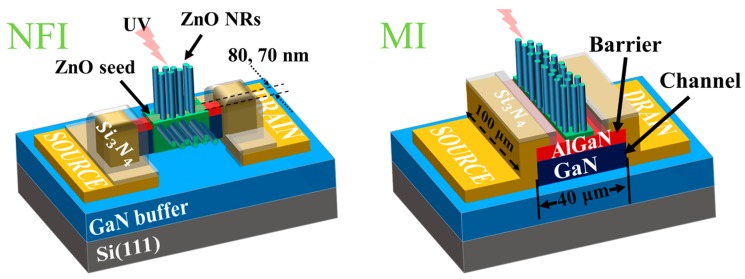
Schematics of the NFI (nanoscale fin isolation) and MI (mesa isolation) structures for the AlGaN/GaN HEMT (high electron mobility transistor)-based UV photodetectors.

Figure 1 shows schematic illustrations of two different HEMT based PD structures examined in this study. The interface of the GaN (channel) and AlGaN (barrier) layers have a confinement of 2-DEG, which acts as channel of very high electron concentration and mobility.

**Figure 2:**
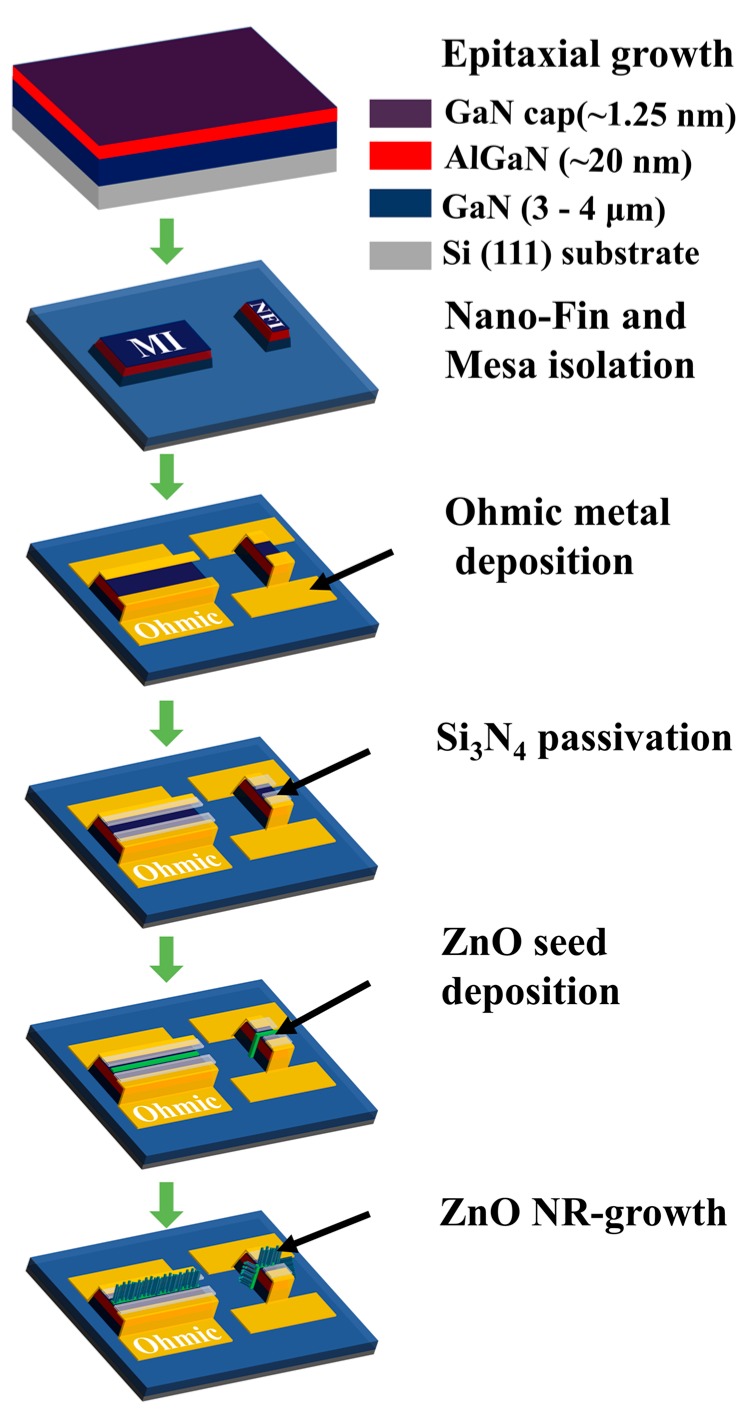
Schematic illustration of the major process steps used for the fabrication of the NFI and MI devices.

Figure 2 illustrates the essential process steps for our gateless NFI and MI HEMT photodetectors (PDs). The epitaxial layers of AlGaN/GaN were deposited on a 6 inch Si (111) by a metal-organic chemical vapor deposition system at NTT-AT (NTT-Advanced Technology Corporation, Kanagawa, Japan). As shown in the schematics, the undoped GaN buffers and channel layers of 3000–4000 nm thickness were grown first, followed by subsequent growths of the barrier layer of ~20 nm Al_0.25_GaN_0.75_ and the GaN cap layer of ~1.2 nm. The measured electron sheet carrier concentration and Hall mobility of the epitaxial layer were ~5 × 10^12^/cm^2^ and ~1750 cm^2^/V·s, respectively. The active area (100 × 40 µm^2^) for the gateless HEMT was defined by a mesa etching of 100 nm depth in the case of conventional MI structures, as shown in Figure 1. The NFI structure, on the other hand, was fabricated with 10 nano-fin-shaped isolations (10 gate fingers) with the same mesa depth of 100 nm and two different fin widths (***W_fin_***) of 70 and 80 nm. By opening the 100 nm Si_3_N_4_ passivation layers deposited on the active region by plasma-enhanced chemical vapor deposition (PECVD), the gate area of a 2 µm gate length was defined. The ZnO NRs were then selectively grown as an active element for the UV light detection.

The epitaxial layers were cleaned and agitated with acetone and ethanol in bath sonication to remove dust and surface contamination, followed by rinsing in deionized (DI) water, then drying with nitrogen (N_2_) gas. Optical lithography (Karl Suss, Garching, Germany, MA6 mask aligner, 365 nm) was used to define the active MI regions, including all the device patterns used in this experiment. However, patterning for the NFIs was performed by an electron beam lithography tool (Jeol, Tokyo, Japan, JBX-9300FS, 100 keV) with a 70 nm PECVD SiO_2_ hard mask to avoid the mask pattern erosion during the mesa etching. After the pattern development of the active regions, mesa etching was performed by removing 100 nm of thickness from the peripheral areas in a reactive ion etching system (RIE, STS Multiplex ICP) using BCl_3_ and Cl_2_ gases. Ohmic contacts were achieved by depositing the metal stack of Ti/Ni/Au (20/30/80 nm), by using an electron beam evaporation system, and a pattern lift-off method, using image reversal photoresist. Ohmic metals were then subjected to a subsequent rapid thermal alloy process at 900 °C for 35 s in N_2_. The samples were then passivated by a 100 nm silicon nitride (Si_3_N_4_) layer deposited in a PECVD system at 200 °C and RF power of 1 kW using a NH_3_/SiH_4_ gas flow rate ratio of 1.5. The gate areas (2 × 100 µm) of two different structures were opened by etching the Si_3_N_4_ passivation using RIE in CF_4_ plasma at a gas flow rate of 110 sccm and a chamber pressure of 40 mTorr (at an etching rate of ~9.4 nm/min) under the RF power of 100 W.

**Figure 3:**
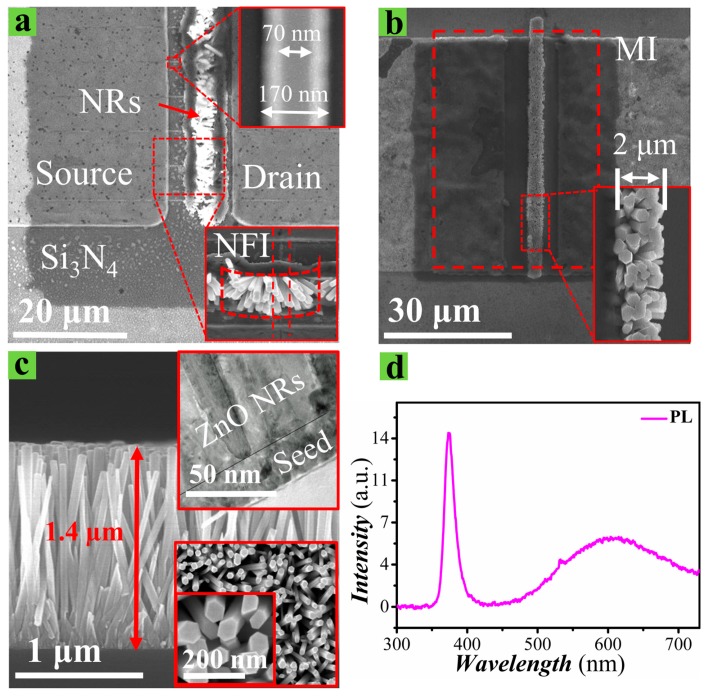
Plane-view SEM images of (**a**) 70 nm NFI PDs (photodetectors) (×1000) with an enlarged top-view of the NFI (***W_fin_*** = 70 nm) (top-right inset, ×5000) and ZnO NR (nanorod) profiles (bottom-right inset, ×3000) and (**b**) MI PDs (×500) with enlarged ZnO NRs profile (bottom-right inset, ×3000). (**c**) Cross-sectional SEM image of the as-grown NRs. Cross-sectional TEM image of the NRs and SL (seed layer) (top-right inset). Top-view of the NRs (bottom-right inset). (**d**) RT (room temperature) PL (photo-luminescence) spectrum of the as-grown ZnO NRs.

ZnO NRs were then grown in the gate area by using the hydrothermal synthesis method. Prior to the growth of NRs, a 20 nm thick seed layer (SL) (as shown in the top-right inset of Figure 3c) was deposited by spin coating the seed solution (3000 rpm and prebaking at 120 °C for 60 s) repeatedly 15 times. After that, the crystalline quality of the SL was improved by annealing the samples at ~350 °C on a hotplate for 1 h. The seed solution used in this study was prepared by dispersing 0.66 g of zinc acetate-dehydrate (C_4_H_6_O_4_Zn·2H_2_O) salt in 30 mL of 1-propanol (CH_3_CH_2_CH_2_OH). In this work, a 20 mM zinc-acetate-dehydrate concentration for seed solution was selected for the desired SL film quality, assuring the ZnO NR crystalline characteristics to be used for this PD application [23]. Various attempts, such as vacuum annealing and O_2_ plasma post-treatment methods [10,24] to improve the SL film quality, which is the key to the high-quality NR crystallites, are also underway in our laboratory. The growth of ZnO NRs was limited to the gate area, which was done by etching the SL from all other areas except the gate region. The samples were then placed in a growth solution for 6 h on a hotplate at ~90 °C to grow ZnO NRs. The growth solution was prepared by mixing 0.25 mole equimolar concentration of zinc-nitrate-hexahydrate (Zn(NO_3_)_2_·6H_2_O, 99%) and hexamethylenetetramine (HMTA) (C_6_H_12_N_4_, 99.5%) in deionized (DI) water. After growth of the the NRs, the samples were carefully cleaned with acetone, ethanol, and DI water, sequentially.

Sloped etching profiles for the NFI structure, the growth morphology of NRs, and the processed device structures were examined by plane-view scanning electron microscopy (SEM, 10 kV S-4800S-Hitachi, Tokyo, Japan). As shown in the top-right inset of Figure 3a, the measured bottom dimensions of the trapezoidal fins were 170 nm after RIE for the ***W_fin_*** of 70 nm. The transmission electron microscope (TEM, 9500-Hitachi) was used to characterize the cross-sectional view and crystalline quality of the as-grown NRs. The RT photo-luminescence (PL) emission spectra from the as grown NRs was obtained by using a He-Cd laser illumination source with a 325 nm wavelength. Gate areas opened with a trench-shaped pattern inside the dark-shaded Si_3_N_4_ passivation and the NRs grown on them are shown in the plane-view SEM micrographs in Figure 3a,b. An inset (bottom-right) of Figure 3a shows the morphology of the ZnO NRs grown around the underlying nano-fins in the gate area of the NFI structures at a magnification of ×3000. Across the gate region, 10 fingers of NFIs are running in parallel, whereas the NRs are grown all the way vertically along the gate area in the MI structure, as shown in Figure 3b. The SEM image in Figure 3c shows that the NRs exhibit an average diameter of ~85 nm and a length of ~1.4 µm. Figure 3d shows a PL spectrum measured at RT from the as-grown ZnO NRs employed as a light absorbing structure in this work. PL characterization at RT is one of the most efficient tools to evaluate the crystalline quality for the WBG ZnO NRs of a direct band gap property. The spectrum exhibits a strong near band edge emission peak at a wavelength of ~380 nm, which is mainly associated with the band-to-band excitonic recombination of ZnO [25]. The near band edge emission intensity was increased about two times with the increase of the NR aspect ratio (AR, length/diameter) from ~8 to ~16. For this reason, NRs with an AR of ~16 were used in this experiment. Emissions in a visible range (420–650 nm) are due to various form of intrinsic defects, such as oxygen vacancy, zinc vacancy, and hydrogen and oxygen interstitials [10]. Despite the inevitable intrinsic defects and consequent visible emissions, as observed from most of the nano-crystallites grown through the aqueous solution based growth methods, our PL spectrum reveals fairly good optical properties in our ZnO crystallites compared to those of the NRs grown using similar methods [2,26,27].

A wide band (300–700 nm) Xenon (Xe) lamp was employed as a light source to measure the photoresponse characteristics of the fabricated devices under optical intensities ranging from 0.5 to 16.5 µW/cm^2^ (100–300 W lamp power). The transient characteristic measurements, according to the UV light (370 nm) on-and-off transient, were carried out by a programmable light shutter controlled in our measurement set-up. The transients of the drain current (***I_ds_***) for the HEMT PDs as a function of time were recorded in a Keithley source measurement unit with a floating gate configuration at a drain voltage (***V_ds_***) of 4 V. The spectral response of the PDs was measured by a focused illumination of a monochromatic light from a wide band (300–1100 nm) Xe-lamp (Ushio UXL-75XE, Ushio Inc., Tokyo, Japan) light source of a 16.5 µW incident optical power. In this measurement set-up, the responsivity was measured at a light chopping frequency of 30 Hz using a lock-in amplifier in a series configuration with drain-source probes for the detection of an amplified change in current. The change of ***I_ds_*** as a function of incident light wavelength varying from 300 to 800 nm was recorded under a floating gate configuration at a ***V_ds_*** of 4 V. To obtain a monochromatic light from the Xe-lamp, a spectral optics monochromator (CM110 ⅛ m) with 2400 lines/mm grating was used.

## 3. Sensing Mechanism

**Figure 4:**
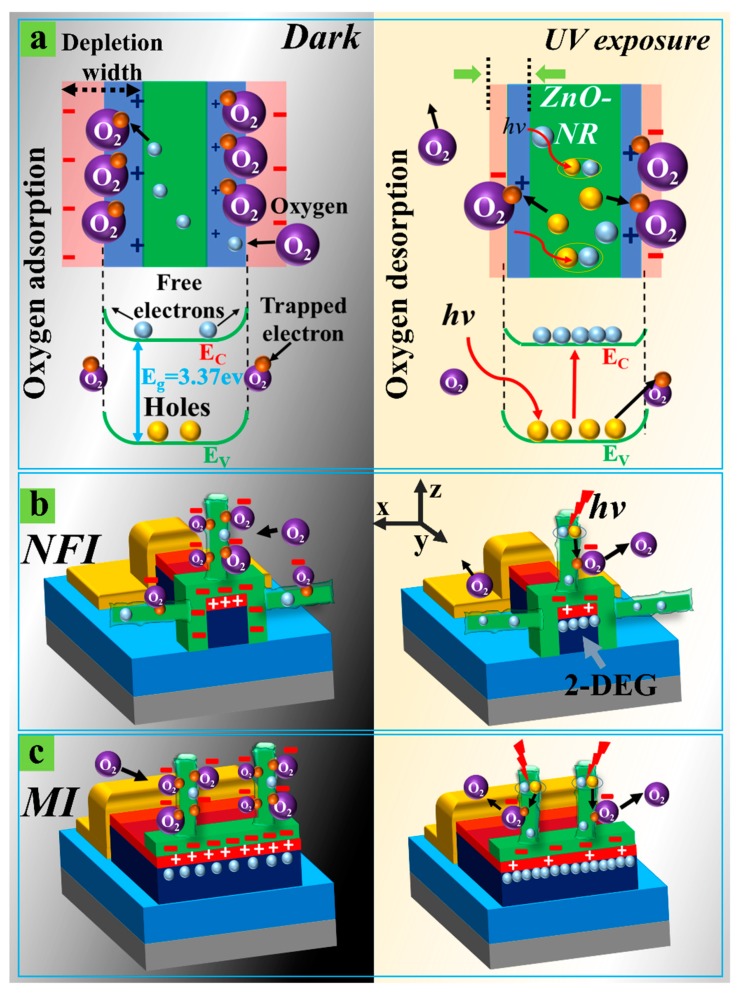
Schematic illustrations of (**a**) oxygen adsorption (left: in the dark) and desorption (right: under UV illumination) at the surface of ZnO-NRs. Oxygen adsorption (left: in the dark) and desorption (right: under UV illumination) processes are taking place at the surface of ZnO NRs grown on the gate areas of (**b**) NFI and (**c**) MI PDs. Cross-sectional schematics of each PD structure viewed in y-axis direction are illustrated; 2-Deg (two-dimensional electron gas).

As illustrated in the schematics of Figure 4, the UV sensing mechanism of the HEMT based PDs depends on the chemisorption of oxygen at the surface of NRs and the consequent change in carrier concentration in underlying 2-DEG. The as-grown crystalline ZnO-NRs are n-type in nature due to a large number of donor defects, such as hydrogen interstitial and Zn interstitial [28]. Under dark conditions (no UV light illumination), oxygen molecules (O_2_) transported from the ambient air to the ZnO NR surface tend to trap electrons from the conduction band of ZnO and leave behind positively charged ionized donors in the surface space charge region, while the negatively charged O_2_ molecules are fixed to the surface of NRs as adsorbed oxygen ions ($O_{2_{\mathrm{ads}}}^{-}$):

(1) $$O_{2}+e^{-}\to O_{2_{\mathrm{ads}}}^{-}.$$

This reaction leads to the expansion of the space charge region near the surface of the NRs due to the depletion of the surface electron states by $O_{2_{\mathrm{ads}}}^{-}$, as depicted in the left of Figure 4a. As a consequence, the adsorption process gives rise to a negative potential at the gate of our HEMT based PDs (as shown in the left of Figure 4b,c), thereby dropping the carrier concentration in the 2-DEG channel at the interface of AlGaN/GaN. This process will eventually reduce the conductance of channel and drain-to-source current (***I_ds_***) in dark conditions. The Schottky gate (Ni/Au) HEMTs (2 µm gate length, 100 µm gate width) fabricated in our group using the same epitaxial structure and Si_3_N_4_ passivation showed a threshold voltage (***V_TH_***) of ~−3 V [29]. This result demonstrates that our HEMT devices with an Au/Ni gate electrode are normally on at zero gate voltage ***V_gs_*** (depletion-mode). We suppose that the ***V_TH_*** of the HEMTs is given by [30]

(2) $$\emptyset_{b}-\Delta E_{c}-\left(en_{s}d^{2}\right)/2\varepsilon$$

where ∅***_b_*** is the Schottky barrier height, Δ***E_c_*** is the conduction band offset, ***e*** is the electron charge, ***n_s_*** is the 2-DEG sheet carrier density, ***d*** is the barrier layer thickness, and ***ε*** is the dielectric constant. Because the difference between the estimated $\emptyset_{b}$ of Ni at the interface with GaN (~1.1 eV) [31] and the GaN surface band bending (~1.0 eV) [32] in ambient air (fully saturated by $O_{2_{\mathrm{ads}}}^{-}$ in the dark state) is quite small, it can be reasonably assumed that a significant amount of dark current (***I_dark_***) through the 2-DEG channel is unavoidable from our gateless MI HEMT devices under a floating gate condition due to their normally-on characteristics as shown in Figure 4c.

On the other hand, a different mode of operation is expected for the NFI structure because the 2-DEG channel region inside the nano-fins is three-dimensionally surrounded by a free surface, whereon the ZnO NRs are grown with many $O_{2_{\mathrm{ads}}}^{-}$ around the surface in dark conditions, as shown in Figure 4b. This makes the NFI PDs operate more closely to a normally-off mode (enhancement mode), thereby exhibiting a much lower ***I_dark_***, because the carriers in the 2-DEG region can be highly depleted, even under the floating gate conditions, due to the surface depletion of the NRs by the $O_{2_{\mathrm{ads}}}^{-}$ surrounding the extremely small volume of the active region. The same phenomenon was also observed in the AlGaN/GaN heterojunction FinFETs [33]. With the decrease of ***W_fin_*** from 200 to 60 nm, it was observed from the HEMT devices that electron density in the 2-DEG channel at zero ***V_gs_*** rapidly drops with a positive shift in ***V_TH_*** because of the fringing-field from the side gates depleting the 2-DEG channel. As a result, the fin-HEMT showed a change in the conduction mechanism from normally-on to normally-off modes.

Due to light absorption, UV light illumination generates electron-hole pairs near the surface of the ZnO NRs [34]. The generated holes recombine with the electrons trapped by $O_{2_{\mathrm{ads}}}^{-}$ at the surface. In this way, the O_2_ molecules start to desorb from the surface, as shown in the right of Figure 4a. This phenomenon gives rise to a reduction of negative charge in the gate region, thereby increasing the carrier concentration in the 2-DEG channel and the drain to the source current (***I_photo_***) under UV illumination, as shown in the right parts of Figure 4b,c. 

As long as the response speed of PDs is accounted for, it can be assumed that the response kinetics upon the UV light on-and-off transient are not controlled by the drift motion of electrons in the 2-DEG channel but by the adsorption and desorption reaction of O_2_ on the ZnO NR surface. This result occurs because the mobility of the electrons confined two-dimensionally in the channel region (~1750 cm^2^ V^−1^s^−1^) [33,35] is so fast that the carrier channel transit time cannot be a bottleneck parameter controlling the whole sensor response speed. As proposed in our previous study, the rate of charge change ***dQ***/***dt*** can be given by [35]:

(3) $$dQ/dt=-\alpha e^{\beta Q}$$

where ***α*** and ***β*** are the constants. By numerically solving Equation (3), it can be determined that one critical parameter affecting the response time is the gate area. From the calculations based on this model, the response (or recovery) time increases with the increase of gate area caused by the consequent increase of the total gate charge. We have much smaller gate capacitance in the NFI PD structure than in the MI structure; therefore, a faster response speed upon UV transient illumination can be expected from the NFI structure.

Spectral responsivity ***R***, which can be defined as a ratio of ***I_photo_*** − ***I_dark_*** to the incident optical power ***P_i_***, is one of the key measures to evaluate the performance of PDs. As reported in our previous studies [19,35], a significant enhancement in ***R*** was shown from the NR-gated PDs due to a vast surface area of the ZnO nanostructure and a much higher surface-to-volume ratio than that of the planar ZnO thin film gate structure. In our HEMT-based PDs, the ***R*** can be also significantly influenced by the gain characteristic for the HEMT, which can be expressed by the change of ***I_photo_*** according to the change of light power irradiating on the gate area.

## 4. Results and Discussion

**Figure 5:**
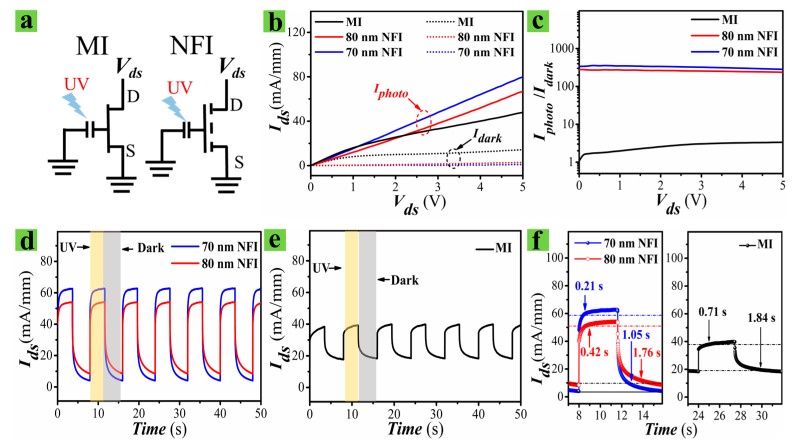
Photoresponse of MI and NFI PDs. (**a**) Equivalent electrical circuits of MI (left) and NFI devices (right). (**b**) Measured ***I_ds_*** versus ***V_ds_*** and (**c**) on-off current ratio (***I_photo_/I_dark_***) of MI and NFI PDs. Time resolved photoresponse characteristics of (**d**) NFI and (**e**) MI PDs (***V_ds_*** = 4 V). (**f**) Magnified views of transient characteristics of NFI (left) and MI (right) PDs.

Figure 5a shows the equivalent electrical circuit diagram of both MI and NFI devices, where MI PD is illustrated as a normally-on transistor while the NFI PD is a normally-off transistor. To examine the characteristic difference of UV responses from the two different PD structures, we first measured ***I_ds_*** in dark conditions (***I_dark_***) and under UV illumination (***I_photo_***) at drain voltage (***V_ds_***), ranging from 0–5 V. UV light exposure was provided from an Xe lamp, operating at 300 W power, with a monochromatic light filter at 370 nm. This UV source produced an incident light intensity of 16 µW/cm^2^, as measured by a power meter. Figure 5b demonstrates that our MI PD exhibits a high ***I_dark_*** of ~10 mA/mm at a ***V_ds_*** of 4 V due to the enhancement-mode operation, as discussed in the previous section. In the case of NFI PDs, significantly reduced values of ***I_dark_*** (~0.19 and ~0.27 mA/mm for ***W_fin_*** of 70 and 80 nm, respectively) were measured at a ***V_ds_*** of 4 V.

The performance of PDs can be evaluated by few important parameters, such as spectral responsivity ***R***, photoconductive gain ***G***, specific detectivity D*, and sensitivity ***S*** [36]. Despite the high ***R*** nature of our MI HEMT-based PDs, this high ***I_dark_*** of the MI structure can critically deteriorate the photo-sensitivity performance associated with ***S,*** which is given by (***I_photo_*** − ***I_dark_***)/***I_dark_***), or the on-off current ratio (***I_photo_***/***I_dark_***), as well as D*. As shown in Figure 5c, much improved on-off current ratios (290~340) were recorded from the NFI PDs compared to those of the MI devices (~4). This result is mainly due to the suppression of ***I_dark_*** caused by the fully depleted 2-DEG channel of the NFI devices in dark conditions.

The high-speed transient characteristic of the UV PD is one of the key factors for real-time application. Specific cases, such as the non-invasive assessment of cancer cells by optical biopsy [37], require ultra-fast PDs with response and recovery time in the order of a few milliseconds. To assess the photocurrent transient of the fabricated devices, the change in ***I_ds_*** as a function of the on-and-off of the UV light exposure time was measured using a programmable shutter. Figure 5d,e shows that both the NFI and MI PDs produce sharp increases in ***I_ds_*** upon UV illumination and a slower fall-off upon termination of UV exposure. The NFI device with a 70 nm ***W_fin_*** showed the fastest rise-up time (or UV response time) and fall-off time (or recovery time) of 0.21 and 1.05 s, respectively. We hereafter define the rise-up and fall-off time as the time intervals for ***I_ds_*** to ramp up to 90% of the maximum saturation value after UV turn-on and to ramp down by 90% from the maximum value after UV-off. On the other hand, the MI device showed much slower response and recovery times of ~0.71 s and ~1.84 s, respectively. This significant improvement in response speed of the NFI device is due to the minimized dimension of gate area where the light absorption takes place; therefore, less time is required to complete the O_2_ adsorption-desorption process in the smaller area of the NFI PDs than in the larger area of the MI device, as discussed in the previous section. The ZnO NR based photoconductive PDs [26,38] still show a long recovery time on a seconds scale, even though a great amount of research effort has been made on enhancing the response speed of these passive PDs. UV PDs based on ZnO nanowire networks with Pt contacts have been fabricated on glass substrates by exhibiting a fast recovery time of 0.2 s with a high photosensitivity (~5 × 10^3^) at 365 nm [39]. The fastest UV PDs of GaN-based metal-semiconductor-metal, p-i-n, or metal Schottky barrier devices [6] have shown extremely high speed (from microseconds to picoseconds) and low-noise capabilities. However, PDs of these GaN-based structures, developed specially to improve the UV response characteristics, exhibit a very low spectral responsivity of less than 1 A/W [8].

**Figure 6:**
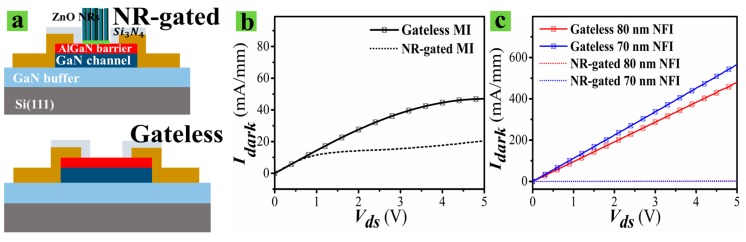
(**a**) Schematic illustration of NR-gated PD and Gateless PD. Measured ***I_dark_*** of MI (**b**) and NFI (**c**) device structure before (gateless) and after NR growths (NR-gated).

Figure 6a shows the schematic illustration of PD before and after the growth of the ZnO NRs. Shown in Figure 6b,c are the measured ***I_dark_*** values of the two different device structures before and after NR growths on the gate region. For the MI PDs, the dark currents were significantly reduced by the attachment of NRs on the gate region. However, the ***I_dark_*** values measured over the entire drain voltage range were still very high (~10 mA/mm) due to the incomplete depletion of the 2-DEG channel in a large volume of the active region. The measured ***I_dark_*** of the NFI PDs before the NR growth were very high, reaching up to ~560 mA/mm, but they rapidly dropped to a very low level of ~hundreds of µA/mm by the attachment of NRs. This result reveals that both the surface depletion effect in the small volume of an active region and the attachment of the NRs with many $O_{2_{\mathrm{ads}}}^{-}$ on their surfaces lead to the formation of a fully-depleted 2-DEG channel in the NFI structure. 

**Figure 7:**
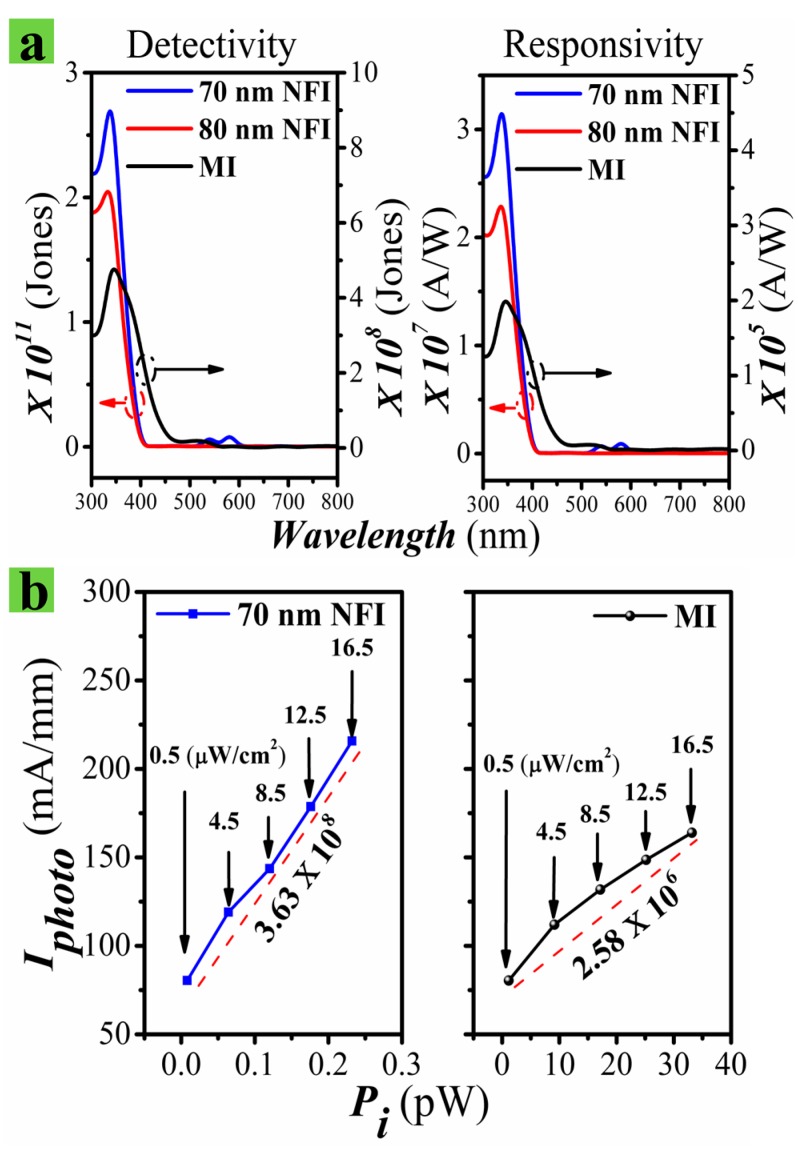
(**a**) Specific detectivity (left) and spectral responsivity (right) as functions of radiant light wavelength measured from NFI and MI UV detectors. (**b**) ***I_photo_*** versus $P_{i}$ of the NFI (left) and MI (right) PDs measured at ***V_ds_*** of 5 V.

The performance of the PDs can be also assessed by spectral responsivity ***R*** and specific detectivity D*, which are expressed in the following equations [36]:

(4) $$R=\frac{I_{photo}-I_{dark}}{P_{i}}$$

(5) $$D*=\sqrt{A}R/\sqrt{2qI_{dark}}$$

where ***A*** is the active area of device, ***q*** is the electron charge, and ***P_i_*** is the radiant light power incident on the active area of the device. The responsivities of our PDs were measured using an incident light intensity of 16 µW/cm^2^ with an effective area of 2.4 × 2.4 mm^2^. NFI PDs showed an extremely high responsivity of ~3.2 × 10^7^ A/W at 340 nm, which is the best performance among any solid-state PDs reported to date [40,41,42,43,44], and even ~100 times higher than that (~2 × 10^5^ A/W at 340 nm) of the MI device, as shown in the right of Figure 7a. This improvement in responsivity of the NFI PD is attributed to a higher photonic transconductance (hereafter, we call it ***g_m,photo_***) characteristic of our HEMT-based PDs, which represents the ratio of the photocurrent change at the drain terminal to the change in incident optical power at the gate terminal. We define the ***g_m,photo_*** as following.

(6) $$g_{m,photo}=\frac{\Delta I_{photo}}{\Delta P_{i}}\left(A/W\right).$$

To calculate the ***g_m,photo_*** values of the two different device structures, the ***I_photo_*** values were measured at various optical intensities, varying from 0.5 to 16 µW/cm^2^ at a ***V_ds_*** of 5 V. Shown in Figure 7b is the measured ***I_photo_*** versus ***P_i_*** from the MI and NFI PDs. The estimated ***g_m,photo_*** values for the NFI and MI PDs were 3.63 × 10^8^ and 2.58 × 10^6^ A/W, respectively. Even though this ***g_m,photo_*** parameter does not directly represent the gain (***g_m_*** = Δ***I_ds_***/Δ***V_gs_***) characteristics of the field-effect transistors, it can be used as a performance measure to estimate the gain characteristic of our gateless HEMT PDs, because the change of incident optical power is directly associated with the virtual Δ***V_gs_*** induced by the change in numbers of $O_{2}^{-}{}_{\mathrm{ads}}$ on the ZnO NR surface under the illumination of UV light.

FinFET technology has recently shown a major increase in adoption of use within Si integrated circuits. The advantages of a FINFET structure, even though there are a number of subtly different forms, can be numerous, but they are basically based on “channel controllability” in a nano-scale channel-length regime of FETs [45]. Furthermore, Si-based FINFETs have shown a significant enhancement in the dependence of ***I_ds_*** on ***V_gs_*** at any applied bias in the sub- and near-threshold regimes by the superior electrostatics of the device architecture [46]. Normally-off Al_2_O_3_/GaN metal-insulator FINFETs (***W_fin_*** = 50 nm, 1 µm gate length) also showed very high maximum ***I_ds_***, ***g_m_***, and maximum field-effect mobility of 1.51 A/mm, 580 mS/mm, and 293 cm^2^V^−1^s^−1^, respectively, due to the more effective increase of 2-DEG electron concentration and higher electron mobility by enhanced gate controllability than the planar devices [47,48]. The superior performance of ***R*** in our NFI PDs is most likely attributed to this enhancement of “gate controllability” screening the field lines effectively from the interface traps or the defects near the 2-DEG channel, thereby reducing the electron scattering in the GaN channel.

The D* of a PD, as defined in Equation (5), is also an important figure of merits used to describe performance. The NFI PDs of 70 nm ***W_fin_*** exhibited a maximum D* of ~3.2 × 10^12^ Jones (cm·Hz^−1/2^/W) at 340 nm, which is four orders of magnitude higher than that of MI PDs (~4 × 10^8^ Jones), as shown in the left of Figure 7a, which is mainly due to the very low ***I_dark_*** of the NFI structure. A very high D* up to a value of 1.4 × 10^15^ Jones [14] was reported from the ZnO NRs based UV PDs. However, the ***R*** achieved by this device was much lower (~10^3^ A/W) than that of PDs.

## 5. Conclusions

Most of the performance parameters, such as ***R***, D*, on-off current ratio, and response speed, were all significantly improved by employing the NFI structure for the AlGaN/GaN HEMT based UV PDs with a ZnO NR UV-absorbing structure. The NFI PD, especially, exhibited an extremely high ***R*** of ~3.2 × 10^7^ A/W. This performance enhancement was due to the subsequent characteristic change of the gateless HEMTs, induced by the reduction of ***W_fin_*** to 70–80 nm. The NFI structure significantly improved the gain characteristics caused by enhanced gate controllability in nano-fin channels beyond the inherent high performance in conversion efficiency of the photon to electron-hole pair generation due to the large surface-to-volume ratio of the ZnO NRs grown in the active region. As the width of the 2-DEG channel is reduced by the NFI profile, the side-wall surface depletion in nano-fins and the attachment of NRs with numerous $O_{2_{\mathrm{ads}}}^{-}$ on the surface of the NRs lead to the formation of a fully-depleted 2-DEG channel and pushed the ***V_TH_*** to a positive value. The measured high on-off current ratio and D* are mainly due to this normally-off operation of the NFI PD structure. An improvement in response speed of the PDs is associated with the minimized dimension of the gate area and the resulting gate capacitance of the NFI structure, where much less time for charging or discharging is required for the O_2_ adsorption-desorption process. The fabricated PDs also showed a linear dependence of photocurrent on the input light intensity in a range of 0.5–16.5 µW/cm^2^, regardless of device structure. The measured ***g_m,photo_*** value for the NFI PDs of 70 nm ***W_fin_*** was 3.63 × 10^8^ A/W, which was ~100 times greater than that of the MI PDs.
